# Myxœdema

**Published:** 1883-11

**Authors:** John A. Robinson


### ARTICLE III.

MYXŒDEMA. By JNO. A. ROBINSON, M.D. Read before the Pathological Society, July 9th, 1883.

We find the first mention of this remarkable and somewhat rare disease in a paper read by Sir William Gull before the Clinical Society of London, October 24th, 1873, entitled: "On a Cretinoid State Supervening in Adult Life in Women." To him belongs the honor of first differentiating the disease. He presented two cases in full, and briefly analyzed five others, in all of which the distinguishing characteristics consisted of a diffused, boggy, swollen condition of the whole body. He did not enter at length into the consideration of the morbid anatomy and pathology of the disease, but was inclined to consider it to be a manifestation of cretinism.

In 1878, Dr. William M. Ord described the same ailment and applied to it the name, "Myxœdema," by reason of the fact that he found, upon two post mortem examinations, a greatly increased deposit of mucin in the areolar tissue. Since that time our literature has been greatly enriched by the observations of both domestic and foreign authors. However, the majority of cases of myxœdema have occurred abroad. So far as I have been able, I find the record of only five cases in this country. These cases were reported by Drs. W. A. Hammond, Allen McLain Hamilion, Holland, Ball and Cashier. Dr. A. Clifford Mercer published quite an exhaustive article on myxœdema in the New York Medical Record of April 16th, 1881.

It has been my fortune to recently obtain the notes of two cases. The first case occurred in the practice of Dr. J. H. Wallace, of Monmouth, Illinois, who kindly sent me the following brief synopsis of the notes of his case: An American wife of a railroad man, æt. 40 years. Had given birth to several children. Menstruated irregularly. Otherwise, in fair health. When first saw her, noticed swelling of face, with glossy appearance of skin. There was a cutaneous eruption, resembling eruption in arsenical poisoning. In fact, this was my first impression as to the trouble, but such was not true. Had scanty urine, bowels inactive, or torpid. No albumen, or tube casts. Numbness of limbs. General œdema, which had existed several months. Did not pit on pressure. Mental processes slow. Speech drawling.

The second case occurred in the practice of Dr. J. P Riss. I examined this patient carefully, and elicited the following history and facts:

Mrs. S.; native of Scotland; aged 52; married; has had seven children, no miscarriages. There is no history of any hereditary ailment.

The patient had always enjoyed geod health until eleven years ago. At that time she noticed she became exceedingly fatigued upon physical exertion. "Her arms and legs became tired," she said. Accompanying this weak bodily state was a correspondingly depressed mental condition. She lost all ambition, and, instead of being energetic, as was usual, she was inclined to take life easily. There was neither melancholia nor hypochondriasis, but merely a lethargic mental condition.

Upon any disturbance of the digestive organs, she was afflicted with a troublesome urticaria. This, however, disappeared upon removing the exciting cause.

Her throat became dry, and deglutition difficult. At the same time there supervened an œdematous condition of the upper and lower eyelids. The swelling gradually extended over face, so that her friends began to remark concerning it. The œdema gradually invaded the whole body, appearing at all parts simultaneously, not at first in dependent parts, or being worse at the end of day, as in dropsy. The skin became dry, glossy, thickened and rough to the touch. Perspiration was infrequent, or absent. Her fingers became so large and clumsy that it was with difficulty she could sew, button her dress, or perform any act requiring dexterity.

For some time she has been afflicted with cold extremities and curious sensations. Her skin has lost its sensibility to a certain degree, and at times she has formications.

Her sense of taste has become impaired, and she does not relish foods. She was formerly very fond of eating. Hearing slightly impaired, while her eyesight has failed quite rapidly recently.

She has never suffered much from pain. Her menopause is over.

At the time I saw the patient she appeared to be a stout woman, however, ill nourished and anæmic. Her gait was unsteady and shambling. She seemed to draw one foot after the other as though there was a loss of muscular power in the limbs. Upon closing her eyes she could stand, or walk, without loss of co-ordination, or the appearance of any ataxic symptoms. During rest the muscles were relaxed.

Her face had a fixed, heavy, almost mournful expression. The features were so changed by the œdema, that the varying emotions could not find expression by the medium of the facial muscles. Her face had a tough, doughy feel. The tongue was enlarged. The œdema of the eyelids was so great as to almost exclude the light from her eyes. She had a peculiar habit of blinking, but there was no photopohobia. The pupils contracted and dilated normally. The conjunctivæ were injected, and lachrymation somewhat profuse.

Her speech was slow and hesitating. On being asked a question, she would hesitate a moment, then answer languidly. The mental processes seemed to be carried on very slowly. Her perceptive faculties were blunted, which fact might account for the slowness of cerebration. The lips were tremulous and bluish.

The bloodless appearance about the eyes contrasted markedly with the blushing tint of the cheeks. The patient was quite subject to flushings, when the color of the face heightened.

Her appetite was capricious, bowels costive and urine scanty. At repeated examinations of the urine the specific gravity was 1020, no albumen, sugar or casts.

She slept pretty well until 2 a. m., each morning, when she awoke and remained awake.

Her temperature was 97° ; pulse 90, small and indicative of increased arterial tension.

There was no discernible disease of any vital organ.

The œdema was not characteristic of dropsy. The swelling was uniform, did not pit on pressure, but the tissues were resilient and imparted to the fingers a tough, doughy sensation.

[illegible] present in the main the points for the diagnosis of this disease. I will recapitulate: The patients are generally adult females. The face is swollen so as to suggest renal disease, which is eliminated by negative signs. The swollen skin is waxy looking and anæmic, and does not pit on pressure. The œdema becomes uniform. The cheeks are overspread with a dull pink flush. Hands lose shapeliness and fingers spade like. In the early stages there is an ever-increasing hebetude involving sensation, voluntary movement, and intellect; in the latter stages aberration of mind often supervenes, the face wears a fixed expression, the speech is slow, voice monotonous, sensation slow but sure. The movements of the limbs are slow and languid; standing requires an effort and falls are frequent. In many cases there is tardiness, but not loss, of co-ordination. The special senses are but slightly affected. The temperature of the body is always below normal. The viscera seldom give signs of disease.

Death comes either by coma, uræmic poisoning or inanition.

I have collected from various sources records of 30 cases, and I find a history of phthisis in two cases, syphilis in two, acute fever in one, pregnancy preceding the disease in two cases. Nearly all of the cases occurred in adult married women, the majority of whom had each borne several children. Only three cases were males. Uterine disease does not seem to figure as a cause of the disease. Albumen was present in twelve of the thirty cases. So far as discovering the cause of the disease is concerned, we must confess our inability to do so at present. Dr. Moravan believes "the disease to be due to a neurosis of the central nervous system, affecting the afferent portion of the motor nerves, both of animal and organic life, they becoming paralyzed." Dr. Mohammed, London, contends that "the disease is due to the chronic œdema of recognizable, or unrecognizable, Bright's disease." The presence of mucin he believes "to be due to an attempt at organization of the chemically œdematous connective tissue." Dr. Ord believes the disease "to be due to the deposit of mucin in the connective tissue." In this way he accounted for the œdema, loss of sensibility of the cutaneous surface and the various nervous phenomena. He believed the mucin acted as a *pad* about the nerves, hindering the propagation of nervous impulses; the central nervous symptoms he believed to be secondary to this hindrance.

Dr. Hamilton, of New York, believes the disease to be of neurotic origin. He says: "Dr. Cashier has found cell degeneration in the giant cells of the anterior horns, as well as the gray matter of the posterior columns. No examination has yet been made of the sympathetic ganglia, and none, so far as I know, of the brain, medulla, or cervical cord—at least no microscopical examination. I believe, however, that when such an examination is made, there will be found lesions of importance. The early appearance of bulbar symptoms and mental disturbance, are, in my mind, suggestive indications that the disease *originates* in the higher center, and not in the cord." The disease then extends to the lateral and posterior columns of the cord, and the cutaneous trophic changes are the result of this extension.

So far, then, are records of four post mortem examinations.

The result has been almost uniform. In all, there was found effusion in the serous sacs. Heart flabby and dilated. Connective tissue everywhere increased in quantity and mucoid in character, its firibrils distinct, nuclei large and distinct, arterials thickened and caliber diminished. Increase in the stroma of liver, kidneys, skin, muscles and spinal cord, the connective tissues in all the cases having undergone the same mucoid retrogression. Dr. Ord, in two cases, found the mucin had increased fifty fold.

The prognosis of myxoedema is very unfavorable. The onward march of the disease is slow but sure. Years pass by and still the patient lives on, however, with the addition of new symptoms. Towards the latter stages of the disease renal trouble supervenes, and the patient rapidly becomes worse. Albumen and tube casts become abundant, the arterial tension is raised, the heart labors more heavily, decided failure of the memory, more or less delirium, and an idiotic aspect of the patient, all indicate that the patient is nearing the end of life. Eventually, with a greatly reduced temperature, the patient dies, comatose, or from inanition.

All treatment has failed to be of any avail. The treatment which is most to be commended is tonic and hygienic. Some have claimed the greatest efficacy for the milk diet; Ballet reporting one case of several years standing in which the milk treatment was followed by a decided amelioration of the symptoms, the skin becoming more supple and the swelling subsiding. Sulphur baths and iodide of potash have been highly recommended.

Dr. Mohammed reports a case in the *Lancet* in which he used nitro-glycerine with the intention of relaxing the arteries, thus reducing the arterial pressure in the capillary circulation. Severe purgations were administered at the same time for the same purpose. Quite a marked improvement took place, but recovery did not ensue. Dr. Hamilton states that in one case he derived much benefit from administering nitro-glycerine, gr. 1-50 three times a day.

In conclusion, I think we may regard myxoedema as a neurotic disease, implicating the trophic and sympathetic nervous systems. I cannot but, also, believe that the lymphatic system is quite at fault. The increase of mucin is doubtless due to the primary invasion of the lateral and posterior columns of the spinal cord, and, also, to a defective action of the lymphatic vessels in performing their functions. The increase of mucin undoubtedly follows defective elimination. We must necessarily regard the disease as retrogressive and fatal.

